# Supplementary material for: Self-organization of vascularized skeletal muscle from bovine embryonic stem cells
Source: bioRxiv. 2024 Mar 25:2024.03.22.586252. Preprint. [Version 1] doi: 10.1101/2024.03.22.586252 (PMC10996461; doi:10.1101/2024.03.22.586252)
Supplement: 6 [file NIHPP2024.03.22.586252V1-supplement-1.pdf]

bioRxiv preprint doi: https://doi.org/10.1101/2024.03.22.586252; this version posted March 25, 2024. The copyright holder for this preprint (which was not certified by peer review) is the author/funder, who has granted bioRxiv a license to display the preprint in perpetuity. It is made available under aCC-BY 4.0 International license.

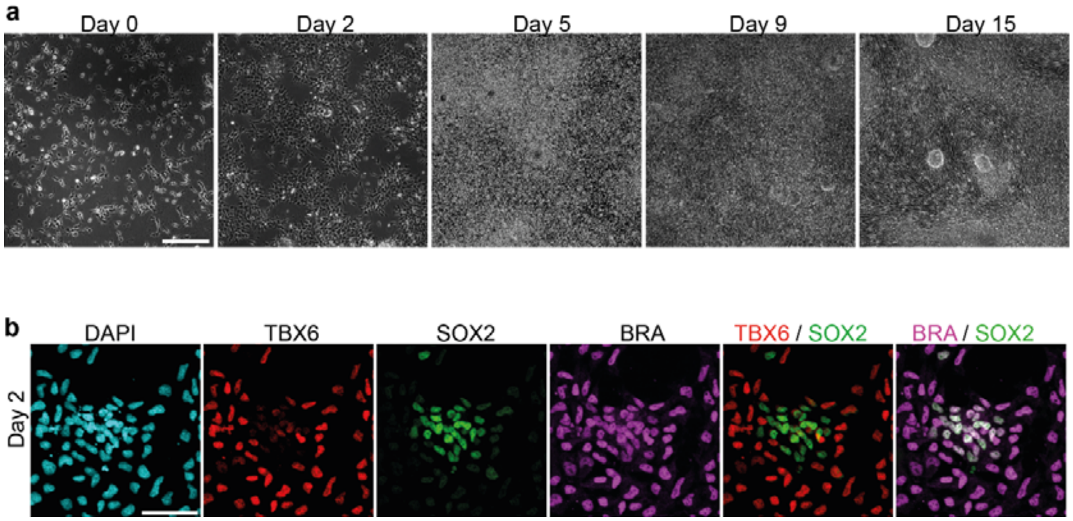

Supplementary Figure 1 Induction of PSM and muscle fibers from bovine ESCs

a, Bright field images over the time course of skeletal muscle induction. The enlarged views of these images are shown in Fig. 1a. **b,** IHC images of the day-2 culture. The enlarged views of Fig. 1b. Scale bars: 300 µm (a) and 50 µm (b).

bioRxiv preprint doi: https://doi.org/10.1101/2024.03.22.586252; this version posted March 25, 2024. The copyright holder for this preprint (which was not certified by peer review) is the author/funder, who has granted bioRxiv a license to display the preprint in perpetuity. It is made available under aCC-BY 4.0 International license.

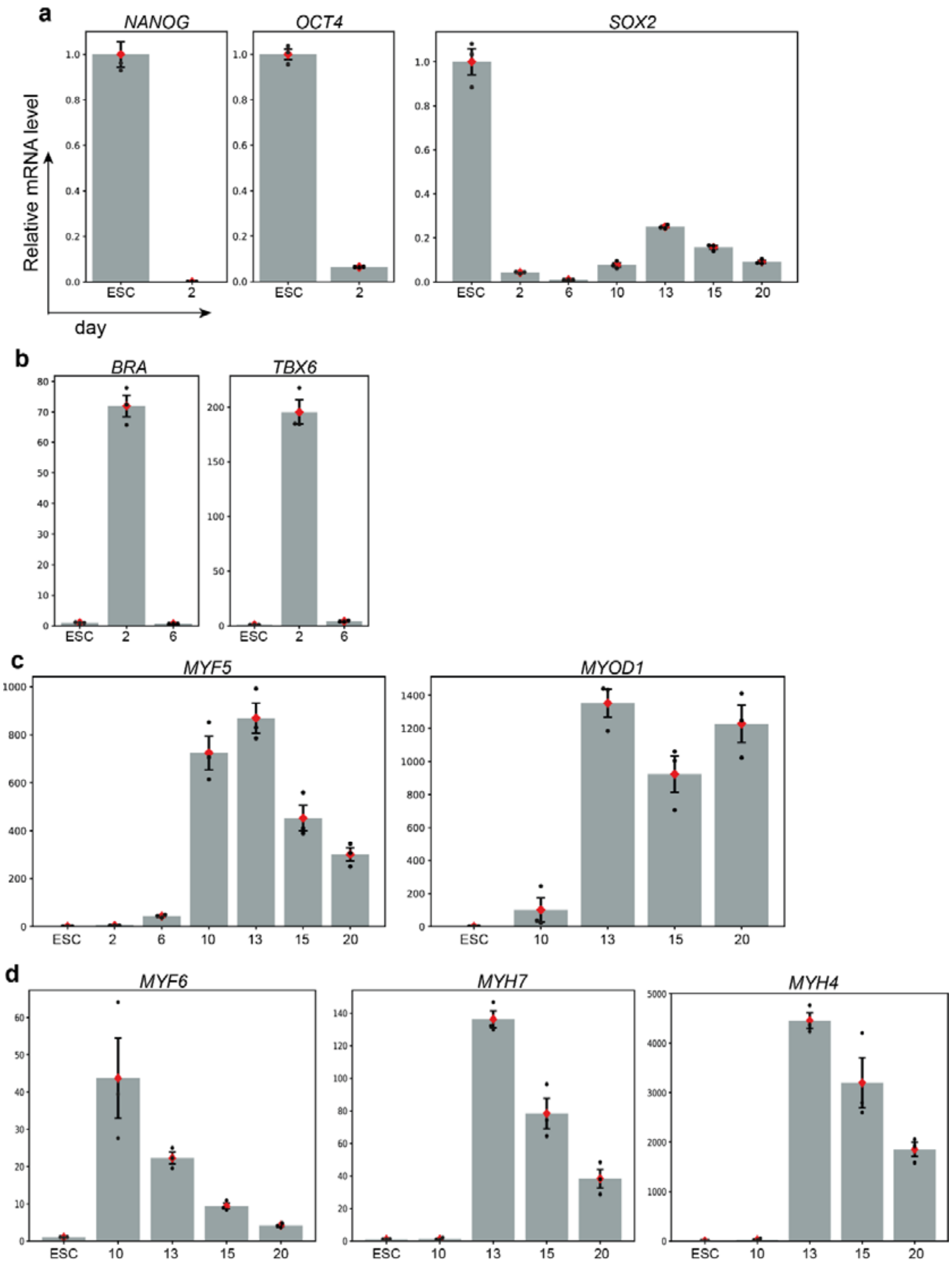

Supplementary Figure 2 qRT-PCR measurements

Relative mRNA levels of selected marker genes in bovine ESCs and the time course muscle induction cultures. The values were normalized to *GAPDH* expression, and the average values of ESCs were set to 1. Mean ± SEM. N = 3 from 3 independent

bioRxiv preprint doi: https://doi.org/10.1101/2024.03.22.586252; this version posted March 25, 2024. The copyright holder for this preprint (which was not certified by peer review) is the author/funder, who has granted bioRxiv a license to display the preprint in perpetuity. It is made available under aCC-BY 4.0 International license.

experiments. **a,** Pluripotency markers. **b,** PSM markers. **c,** Myoblast markers. **d,** Myocyte markers.

bioRxiv preprint doi: https://doi.org/10.1101/2024.03.22.586252; this version posted March 25, 2024. The copyright holder for this preprint (which was not certified by peer review) is the author/funder, who has granted bioRxiv a license to display the preprint in perpetuity. It is made available under aCC-BY 4.0 International license.

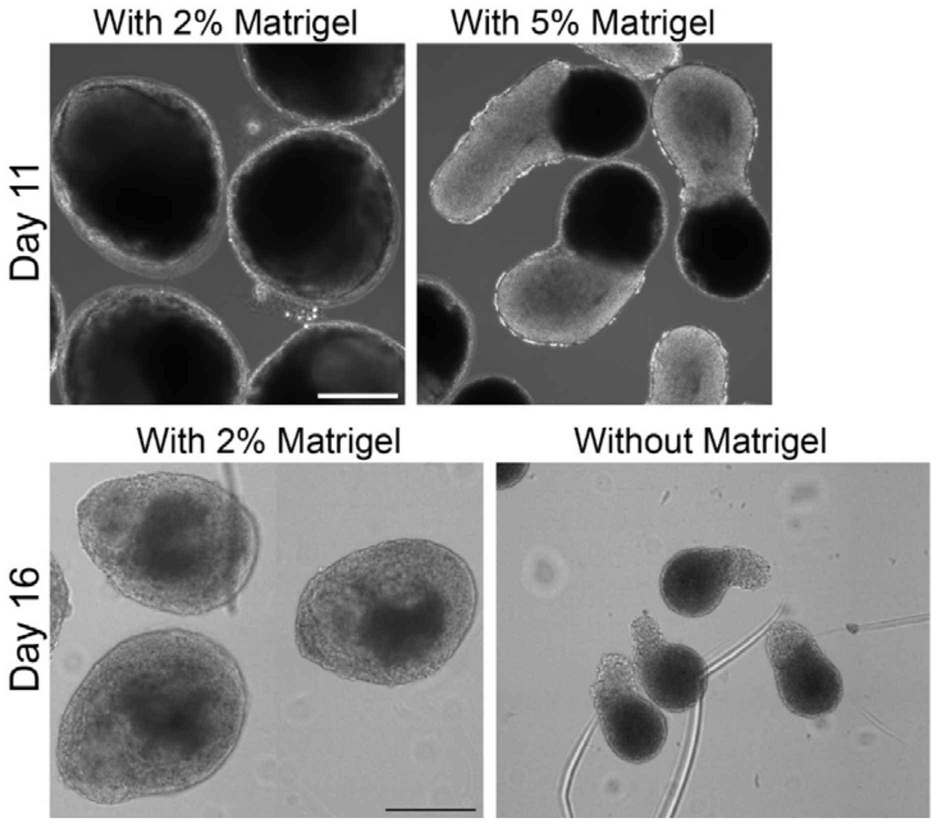

Supplementary Figure 3 Effects of Matrigel on aggregates

Bright field images of the muscle aggregates made without or with (2% or 5%) Matrigel in the medium. 2% is the standard concentration. 3 independent experiments showed similar patterns. Scale bars: 300 μm.

bioRxiv preprint doi: https://doi.org/10.1101/2024.03.22.586252; this version posted March 25, 2024. The copyright holder for this preprint (which was not certified by peer review) is the author/funder, who has granted bioRxiv a license to display the preprint in perpetuity. It is made available under aCC-BY 4.0 International license.

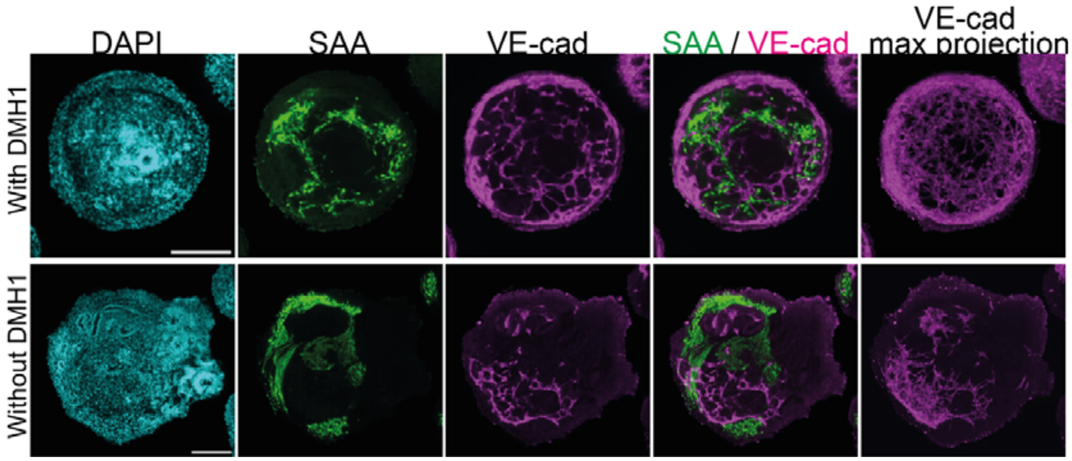

Supplementary Figure 4 Effects of BMP inhibitor during PSM induction

IHC images of the day-15 aggregates. The aggregate made with the standard PSM medium with DMH1 (the BMP inhibitor) showed a uniform VE-cad-positive endothelial network throughout the tissue (top), while the one made without DMH1 showed local endothelial domains (bottom). The max projection of VE-cad used the Z-stacks within the 40 μm range around the central slice. 5-10 independent experiments showed similar patterns. Scale bars: 200 μm.

bioRxiv preprint doi: https://doi.org/10.1101/2024.03.22.586252; this version posted March 25, 2024. The copyright holder for this preprint (which was not certified by peer review) is the author/funder, who has granted bioRxiv a license to display the preprint in perpetuity. It is made available under aCC-BY 4.0 International license.

Name	5'-3'	bp	Tm	
NANOG_Fwd	AGTTACGTGTCCTTGCAAAC	20	61.7	Original
NANOG_Rev	TGTGGAAGAATCAGGACTGT	20	61.7	Original
OCT_Fwd	CAAATTAGCCACATCGCC	18	59.6	Wu X. et al (Sci Rep 2016, 6, 28343)
OCT_Rev	AGCCTCAAAATCCTCACG	18	60.2	Wu X. et al (Sci Rep 2016, 6, 28343)
SOX2_Fwd	TCAGATGCAGCCCATGCAC	19	66	Wu X. et al (Sci Rep 2016, 6, 28343)
SOX2_Rev	GGTGCCCTGCTGAGAATAGGAC	22	67.6	Wu X. et al (Sci Rep 2016, 6, 28343)
BRA_Fwd	TGATCACCAGCCACTGCTTC	20	65.5	Wu X. et al (Sci Rep 2016, 6, 28343)
BRA_Rev	CCTTTGCATCGAGGAAAGCTTT	22	64.7	Original
TBX6_Fwd	GAACAGAGATGATCATCACCAAA	23	61.6	Original
TBX6_Rev	ACGTCCAGAAGAAACAGGTA	20	61.6	Original
MYF5_Fwd	AGGGAACAGGTGGAAAACTA	20	61.7	Original
MYF5_Rev	TCTTTCTGGACCAGATAGGG	20	61.3	Original
MyoD1_Fwd	CGAACACTACAGCGGCGACT	20	67.5	Original
MyoD1_Rev	CGCTGTAGTAAGTGCGGTCGT	21	67.3	Original
MYOG_Fwd	CCAGTACATAGAGCGCCTGC	20	65.5	Original
MYOG_Rev	GTTGGGGCCAAACTCCAGTG	20	66.6	Original
MYF6_Fwd	GGATCAGCAGGACAAAATGC	20	62.5	Original
MYF6_Rev	CCTGGAATGATCCGAAACAC	20	61.6	Original
MYH7_Fwd	CCAGGTGTTGCAGCAAAACC	20	65.5	Original
MYH7_Rev	GGTTGATGGTGACGCAGAAGA	21	65.6	Hu X. et al (Animals, 2019, 9, 859)
MYH4_Fwd	CTCCTAATCACCACCAACCCATA	23	64.7	Hu X. et al (Animals, 2019, 9, 859)
MYH4_Rev	GCCCCTGTGAGCTTGTAGAT	20	64.8	Original
GAPDH_Fwd	ATGACCACTTTGGCATCGTG	20	63.9	Original
GAPDH_Rev	CAGGGATGATATTCTGGGCA	20	62.3	Original

Supplementary Table 1

Primer sequences for qPCR.

bioRxiv preprint doi: https://doi.org/10.1101/2024.03.22.586252; this version posted March 25, 2024. The copyright holder for this preprint (which was not certified by peer review) is the author/funder, who has granted bioRxiv a license to display the preprint in perpetuity. It is made available under aCC-BY 4.0 International license.

Supplementary Video 1

Time-lapse imaging of calcium level fluctuations in the day-18 culture using Fluo-8 in the absence (left) and presence (right) of 10 μM curare. The time stamp indicates min:sec. The snapshot is also shown in Fig. 2a. Images were taken with the Thunder Imager Live Cell & 3D assay.

Supplementary Video 2

Z-stack IHC images of the day-15 aggregates from the 3D muscle and neuron co-induction protocol. SAA and TUJ1 are muscle fiber and neuronal markers, respectively. Z-stack interval is 2 μm. The snapshot is also shown in Fig. 4d. Images were taken by a MuVi-SPIM Light-Sheet Microscope.

Supplementary Video 3

Z-stack IHC images of the day-15 aggregate from the 3D muscle and endothelial co-induction protocol. SAA and VE-cad are muscle fiber and endothelial markers, respectively. Z-stack interval is 2 μm. The snapshot is also shown in Fig. 5d. Images were taken by a MuVi-SPIM Light-Sheet Microscope.

Supplementary Video 4

Z-stack IHC images of the day-15 aggregate from the 3D muscle and endothelial co-induction protocol. ZO-1 is a tight junction marker. Z-stack interval is 2 μm. The max projection is also shown in Fig. 5g. Images were taken by a MuVi-SPIM Light-Sheet Microscope.

Supplementary Video 5

Z-stack IHC images of the day-15 aggregate made from the PSM cells that were induced without BMP inhibitor DMH1. Z-stack interval is 2 μm. The snapshot is also shown in Supplementary Fig. 4. Images were taken by a MuVi-SPIM Light-Sheet Microscope.
